# Long‐Term Regime Shifts in Xeric Ecoregion Freshwater Fish Assemblages due to Anthropogenic and Climate Stressors

**DOI:** 10.1002/ece3.72067

**Published:** 2025-09-01

**Authors:** Corey A. Krabbenhoft, Jane S. Rogosch, Freya E. Rowland

**Affiliations:** ^1^ Department of Biological Sciences University at Buffalo North Campus Buffalo New York USA; ^2^ U.S. Geological Survey, Texas Cooperative Fish & Wildlife Research Unit and Department of Natural Resources Management Texas Tech University Lubbock Texas USA; ^3^ U.S. Geological Survey, Columbia Environmental Research Center Columbia Missouri USA

**Keywords:** Anthropocene, arid, desert, fish assemblages, long‐term data, multiple stressors, rivers, streams

## Abstract

Shifting climate regimes are projected to increase the area of xeric regions and result in more pronounced intermittency across river networks. Given these projected changes, we aim to understand the factors contributing to species persistence under increasing aridity. To investigate how changing flow regimes are related to changes in fish richness and assemblage composition, we compiled data from 1473 xeric stream sites in the United States and Australia. The temporal coverage of this dataset is more than 40 years, from 1980 to 2021. Our focus was on fishes occurring in xeric streams and included 191 species. We compiled climate, hydrologic, and fish species trait data to identify relationships between environmental drivers of species persistence and corresponding characteristics common to species in these systems and traits eliciting the strongest responses to environmental change. Our data show declines in overall precipitation in concert with increasing temperatures over the last several decades. Climatic shifts were accompanied by declines in discharge, increased zero‐flow days, and longer durations of no‐flow periods. In these same systems, an overall linear decline in fish species richness was observed, but it was not directly correlated with any hydrologic predictors. However, xeric species of conservation concern were small‐bodied and occupied lower trophic levels than those not of concern. Listed species were primarily affected by multiple stressors, including habitat degradation and invasive species, compounded by a small geographic range. We thus propose a multiple stressors argument for the declines in xeric fish assemblages, something that may be exacerbated by climate alterations in the future. This work highlights a critical conservation need for xeric fishes and identifies taxa that are especially vulnerable to a combination of anthropogenic stressors and changing climates.

## Graphical abstract

We aim to understand the factors contributing to the persistence of freshwater fishes under altered climate and flow regimes and inform the long‐term conservation of global fish biodiversity. We analyzed changes in fish richness and assemblage composition in xeric streams from 1473 sites in the United States and Australia. In systems where climatic and hydrologic shifts have occurred, an overall decline in fish species richness was observed, with the most pronounced changes in small‐bodied species with limited ranges that occupy lower trophic levels and that are also affected by anthropogenic factors including habitat degradation and invasive species. General declines in fish species richness were observed in the United States where the longest datasets of fish and corresponding flow data were available.

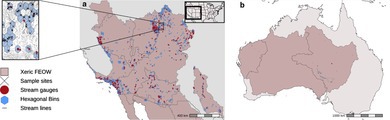

## 1 Introduction

Xeric ecoregions, those with limited surface water supply located in arid, semi‐arid, or dry sub‐humid climates, cover over 40% of the Earth's land surface (Gaur and Squires 2018) and shifting climate regimes are projected to increase the area of these regions globally (Feng et al. 2014). Successively warmer temperatures, as witnessed over the past 40+ years, and intensification of the hydrologic cycle (Gu et al. 2023) will likely contribute to increased aridity worldwide. Consequently, stream intermittency, which currently occurs across more than 50% of global stream length (Messager et al. 2021), is anticipated to increase in prevalence and duration as the full effects of climate change are realized. For example, approximately 59% of streams and rivers in the continental United States are intermittent, but that figure is 80% for streams in the xeric southwestern United States (Goodrich et al. 2018); projections indicate potential increases in the duration of zero flow periods where there is no measurable streamflow (Zipper et al. 2021). Analogous work has been conducted in Australia, where xeric ecoregions are equally expansive. For example, arid and semi‐arid climates characterize over 70% of the Australian continent (Fujioka and Chappell 2010), and this proportion is expected to increase, expanding into once humid, subtropical coasts (Feng et al. 2014). Changes in climate regimes induce fundamental alterations of emergent properties in aquatic systems (Staudinger et al. 2021) and understanding organismal response to these shifts in key locations with relevant long‐term data sets, like the United States and Australia, is a key component of preparing for a more arid future.

In xeric ecoregions, increases in stream intermittency may have pronounced effects on obligate aquatic taxa that are already occupying water‐stressed conditions (Crabot et al. 2021). Many intermittent tributaries are important for spawning, rearing, foraging, refuge, and dispersal (Cathcart et al. 2018; Colvin et al. 2019; Heim et al. 2019). Likewise, xeric fishes rely heavily on perennial refugia (areas of the stream network that do not periodically dry) during periods of intermittency in dry seasons or during droughts (Davis et al. 2013; Magoulick and Kobza 2003). Given that temporary water drives connectivity, which is integral for maintaining gene flow and genetic diversity among these populations (Chafin et al. 2019) and phylogeography of xeric‐adapted fish (Mossop et al. 2015), metacommunity connections that support assemblages may be in jeopardy from the projected increases in the spatial extent of intermittency. Examples of this phenomenon have already been identified in the Murray‐Darling basin, where limited access to refuge habitat due to connectivity losses during low flow conditions has been identified as a major concern for the persistence of fish assemblages (Koehn et al. 2020).

While increasing intermittency poses a considerable spatial challenge for water availability, the resilience of fishes to ongoing anthropogenic and ecosystem stressors of growing water demands and climate change remains uncertain (Lennox et al. 2019; Gido et al. 2023). Xeric fish populations are well adapted to seasonal patterns in hydrology, with population booms in periods of high stream connectivity (Arthington and Balcombe 2011). Fishes in xeric ecoregions have evolved traits that allow them to better tolerate stresses associated with flashy hydrology, high temperatures, and high solar radiation (Logue et al. 1995; Mims and Olden 2012; Shcherbakov et al. 2013). However, rising temperatures, increased intermittency, and reduced flow have resulted in reduced assemblage richness and density in xeric rivers (Gido et al. 2019), perhaps due to the exceedance of physiological thresholds associated with temperature and drying extremes (Sandblom et al. 2016). Intermittency can also compound existing stressors. For example, the combination of severe drought, followed by intense rain and flooding, has contributed to hypoxic blackwater events resulting in substantial fish kills when flows return (Whitworth et al. 2012). Studies further indicate native xeric fishes are more sensitive to interannual variation in climate than nonnative fishes, and alterations of drying regimes have led to species replacements with invasive competitors (Gido et al. 2019; Ruhí et al. 2016). Additionally, reduced streamflow lowers diversity and changes the composition of the riparian invertebrate community, which is a major food source for several freshwater fishes (Allen et al. 2024). Collectively, these findings indicate that intensified intermittence regimes are expected to affect water quality, community composition, and resource availability for xeric fishes, particularly in areas where these effects may be compounded by other anthropogenic stressors. Climate change may especially challenge endemic fish species isolated to small geographic regions (Jaeger et al. 2014), and the occurrence of endemism is higher in xeric fishes (Oberdorff et al. 1999). Stream fish vulnerability to climate change and habitat alteration is a function of environmental tolerances, rarity, range size, dispersal ability, and the connectivity of streams they occupy (Sievert et al. 2016). Increased periods of low or no flow associated with water abstraction and diversion for anthropogenic use (Datry et al. 2023) further exacerbate these challenges. Additionally, trait synergies (e.g., traits associated with slow life cycles) increase the susceptibility of native fishes to rarity, extinction, and extirpation (Olden et al. 2008).

Given observed and projected changes in xeric stream and river systems, it is increasingly important to understand the factors contributing to the success or failure of species to persist under these conditions. This work aims to shed light on the relationship between long‐term changes in hydrologic conditions and xeric fish assemblages and to determine whether changing climates are the major driver in conservation status or species loss. We hypothesized that broad‐scale changes including reduced stream flow, increased temperatures, and decreased precipitation would negatively affect xeric fishes. We compiled data on 183 fish species collected from 1473 sample locations between 1980 and 2022 in the United States and Australia—two areas with substantial xeric ecoregion space—to explore how widespread changing climate and flow regimes are affecting xeric fish assemblages in combination with other anthropogenic stressors. Additionally, we compiled trait data, including body size, longevity, and diet, to identify fish functional traits that influence resistance and resilience to climatic and hydrologic changes. We compiled these data with the goal of understanding broad‐scale patterns of persistence in xeric fishes of the United States and Australia under increasing pressures of climate change compounded by anthropogenic stressors.

## 2 Methods

### 2.1 Fish Data

We conducted a search for global fish occurrence data, with a focus on areas incurring repeated sampling events (Table 1). Following initial efforts, we narrowed our search to the United States (USA) and Australia (AUS) due to limited publicly available data in other regions. Prior efforts to compile fish occurrence data (Comte et al. 2021) aided our efforts, and we supplemented this with a complementary search in the Global Biodiversity Information Framework (GBIF.org 2023) and data from the Atlas of Living Australia (ALA 2023). Fish data were spatially filtered (refer to “Spatial Filtering” below) to only include species that occupied xeric freshwaters, and only sampling points where the majority of species were freshwater were included in our analyses. Data were evaluated for duplicates, and data points with missing information (e.g., coordinates) were removed. To standardize scientific names, we conducted a biological names review using the Integrated Taxonomic Information System, which provides a current literature‐referenced and expert‐validated digital taxonomy of species (ITIS 2023). In rare cases where names were not found in ITIS—10 out of 399 species—we cross‐referenced with FishBase (Froese and Pauly 2022) to confirm the genus and species. Resolution of the *Gila robusta* complex is still debated (Chafin et al. 2021) but given recent naming convention updates (Committee on Names of Fishes 2023), we kept *Gila nigra* , *Gila intermedia* , and *Gila robusta* as separate species in our dataset (i.e., headwater chub, Gila chub, and roundtail chub).

**TABLE 1:** Fish occurrence, stream discharge, and climate data sources and sample sizes following spatial filtering for overlap with xeric ecoregions and removal of primarily marine sites and species.

Data type	Data source	Date range	Number of sites	Sampling events	Source reference
Fish occurrence	Atlas of Living Australia	1924–2022	171	625	Atlas of Living Australia (2023)
Fish occurrence	GBIF	1954–2021	4272	32,922	GBIF (2023)
Fish occurrence	RivFishTime	1975–2018	52	7082	Comte et al. (2021)
Total fish occurrence		1924–2022	4495	40,629	
Australian stream gauges	CAMELS, GRDC	1979–2021	34	Daily average discharge	Fowler et al. (2020) GRDC (2021)
USA stream gauges	USGS	1979–2021	162	Daily average discharge	U.S. Geological Survey (2016)
Total stream gauges			196		
Temperature data	NOAA	1979–2022	1362	Interpolated daily average	NOAA (2023)
Precipitation data	NOAA	1979–2022	1378	Interpolated daily accumulation	Xie et al. (2007) and Chen et al. (2008)

*Note:* These raw data were spatially binned by hexagon into 1473 spatially explicit sampling areas.

Abbreviations: CAMELS, Catchment Attributes and Meteorology for Large‐sample Studies; GBIF, Global Biodiversity Information Framework; GRDC, The Global Runoff Data Centre; NOAA, National Oceanic and Atmospheric Administration.

For each verified fish species in the resulting dataset, we collected trait data using a combination of FishBase (Froese and Pauly 2022), the fish traits database (Frimpong and Angermeier 2009), freshwaterecology.info (Schmidt‐Kloiber and Hering 2015), Fishes of Australia (Bray and Gomon 2022), and the International Union for Conservation of Nature and Natural Resources (IUCN 2022). We chose to examine traits that had continuous data, few missing points, and their relevance to persistence in xeric environments. We examined endemism (which we defined as only occurring within one drainage area of a major river, equivalent to a HUC2 water resource region, using the U.S. Geological Survey (USGS) classification system), estimated trophic level (internal FishBase calculation), maximum total length (cm), longevity (maximum age in years), IUCN status, and the reason(s) for IUCN listing.

### 2.2 Spatial Filtering

**FIGURE 1:**
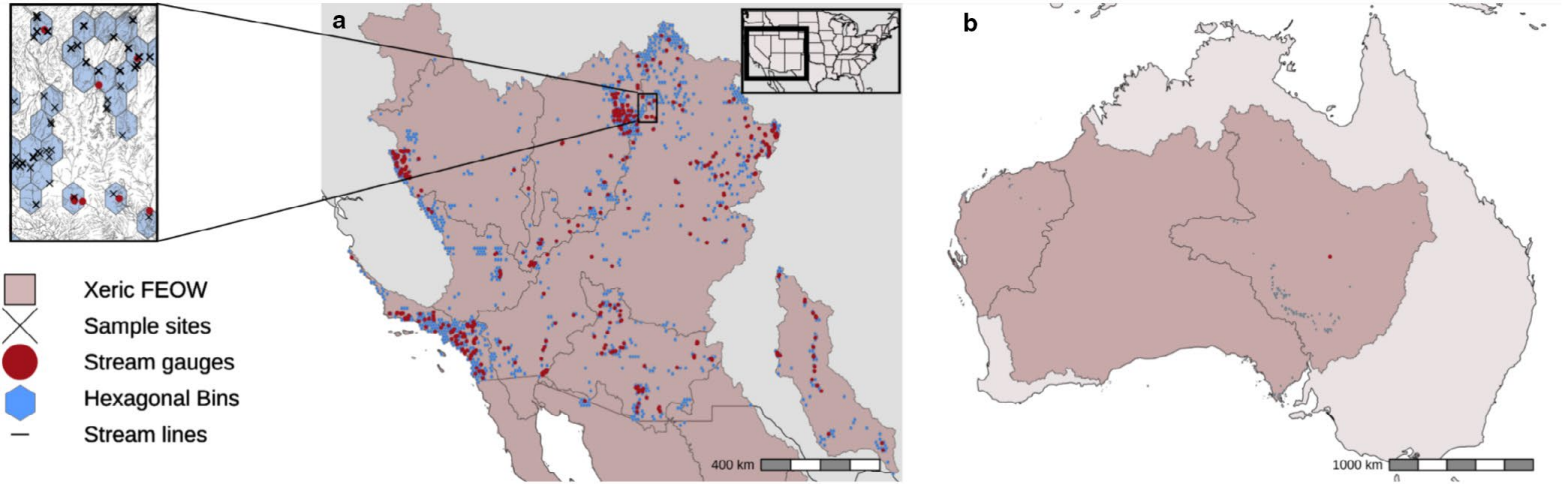
Final data layers following spatial filtering, where discharge and fish data overlap in xeric ecoregions. Maps show the southwestern United States (a), and Australia (b). Overlap with xeric Freshwater Ecoregions of the World (FEOW; https://feow.org/) is shown in shaded polygons, fish occurrence locations are noted by ‘X', spatially relevant stream gauge locations are red points, and sampled hexagonal bins are shown in blue. The inset map shows an example of hexagonal binning of sampling locations and stream gauges relative to the stream network.

To address questions tailored to xeric streams, all fish location information was spatially filtered for overlap with Xeric Freshwaters and Endorheic Basin habitat areas per the Major Habitat Types designated by Freshwater Ecoregions of the World (FEOW; Abell et al. 2008). Data were evaluated for the number of digits included in the latitude and longitude (decimal degrees) to determine the level of spatial error. Given our spatial approach using hexagonal bins, a minimum of three digits was deemed appropriate (e.g., 46.1, −99.4). The distance between 1/10 decimal degrees should be less than a 150 km radius of error and would fit well within the hexagonal bins we use to concatenate fish occurrence data. Of the final list of fish occurrence data used, 2949 instances only had two digits for latitude (all longitudes had at least 3 digits), all of which had either five or nine digits in the longitude value and so were assumed to include zeroes as significant digits for the latitude. These values were thus assumed to be specific to site location and kept for further processing. Data were further filtered to include only those where at least 50% of the species occurrence data were from freshwater species (including migratory taxa) to remove those that were mostly coastal assemblages outside of the target xeric stream network; any marine‐ or brackish‐specific taxa (that potentially move in and out of freshwater habitats) were additionally excluded as outside the scope of our research questions. Because of the patchy nature of fish sampling and the multiple sources for fish occurrence data, fish sampling sites were spatially binned to identify sampling locations, climate data, and stream gauges that likely represent the same local assemblage and conditions; this approach increased our ability to look at whole‐assemblage compositions through different sampling strategies and increased the temporal resolution of data to address trends over time. The hierarchical, fractal nature of stream networks does not lend itself to simple spatial clustering approaches, so we used a hexagonal grid approach to bin sites within hex‐cells (noted as HexIDs) with a 0.1 decimal degree radius (centroid to vertex), which corresponds to approximately 11 km and produces a binned area of 314 km^2^ (Figure 1).

### 2.3 Climate Data

Global climate data were obtained from the Climate Prediction Center's Global Unified Temperature (NOAA 2023) and Global Unified Gauge‐Based Analysis of Daily Precipitation datasets (Chen et al. 2008; Xie et al. 2007). The data represent modeled information from 1979 to 2022 from a 0.5°x0.5° global grid. Climate data were extracted from the coordinates of each hexagonal cell centroid to provide insight into precipitation and temperature changes over the period of interest with specific relevance to local conditions at the sites where fish occurrence data were compiled. Daily maximum and minimum temperatures (°C) were extracted from krigged data from the global grid and used to calculate daily averages. Precipitation was represented by daily totals (in mm). Temperature and precipitation data were further trimmed to include only sites with records extending the entire period of interest, which excluded 111 and 95 hexagonal bins, respectively (final temperature data: USA *n* = 1329, AUS *n* = 33 sites; final precipitation data: USA *n* = 1338 sites, AUS *n* = 40 sites).

We calculated metrics separately for each hexagonal cell. Annual precipitation metrics were calculated for each year in the dataset and included total precipitation, precipitation anomaly, total zero precipitation days, and precipitation intensity. We calculated annual precipitation as the sum of all daily precipitation within a calendar year defined as 1 January to 31 December. Annual precipitation anomaly was calculated by subtracting the long‐term average for each location from the yearly average and was evaluated as a continuous variable, where more negative values indicated lower than average precipitation and more positive values indicated higher than average precipitation. Zero precipitation was the sum of days without any measurable precipitation (< 1 mm) in a calendar year. Precipitation intensity was the total annual precipitation divided by the number of days in that year with rain. We additionally calculated temperature metrics by season and included average seasonal temperature and temperature anomaly (calculated as above). In Australia, spring was defined as September to November, summer as December to February, fall as March to May, and winter as June to August. In the United States, spring was defined as March to May, summer as June to August, fall as September to November, and winter as December to February.

### 2.4 Discharge Data

To evaluate changes in water budgets across the time periods where fish sampling occurred, discharge data were obtained from several sources to acquire spatial representation for the USA and AUS (Table 1). Data for the USA was obtained from U.S. Geological Survey surface water data for the nation via the USGS ‘dataRetrieval’ package in R (De Cicco et al. 2024; R Core Team 2023). Data for Australia came from compiled global datasets (Fowler et al. 2020; GRDC 2021). Stream gauges were spatially binned using the same hexagonal‐grid approach as outlined above. For USA gauge data, stream gauges were filtered to only include gauges where spatially filtered fish occurrence data occurred within the same hexagonal grid cell to provide some insight into the influence of hydrology on fish occurrence (gauge *n* = 162). Only a single gauge from Australia was contained within a hexagonal cell containing fish occurrence data, so all long‐term gauges in the xeric ecoregion (*n* = 34) were used to provide insight into regional trends. To examine drying patterns, we calculated flow metrics specifically designed to investigate the intermittency signature of streams (Hammond et al. 2021; Zipper et al. 2021). For each cell, we calculated the maximum total number of zero‐flow days per water year, the incidence of the first day of minimum flow, and the maximum duration (in days) of any single zero‐flow period. Zero‐flow is defined as a zero discharge measurement recorded at any gauge within the cell. To more accurately capture the zero‐flow conditions of interest, we used stage height to differentiate between flow reversals and dry stream channels (Zimmer et al. 2020). To examine annual trends, we used mean normalized and log‐10 transformed discharge data to calculate daily flow anomalies according to a discrete Fast Fourier Transform (Sabo and Post 2008; Shah and Ruhi 2019) for each gauge and then summed daily values to calculate the net annual anomaly (flow anomaly or NAA) for each gauge. Flow anomaly was evaluated similar to precipitation anomaly, with a value of zero indicating a year with average flow.

### 2.5 Trends in Precipitation, Temperature, and Stream Discharge

To identify climate trends as they relate to the water budget across years in our study, we analyzed trends in precipitation, temperature, and discharge from 1980 to 2021 in R v4.3.1 (R Core Team 2023). To explore the seasonal trends in precipitation and temperature across the entire regions of AUS and USA, we used the Kendall trend test in the R package ‘wql’ (Jassby and Cloern 2024) to explore how monthly precipitation trends for the period of interest at 127 sites in Australia and 1346 sites in the USA have changed. This package is an extension of the Mann‐Kendall test for trends that accounts for common issues such as nonnormality of data, serial dependence, and seasonality (Hirsch et al. 1982), and is intended for datasets with monthly data at discrete stations. We used ‘seaKen’ in conjunction with ‘mts2ts’ to calculate a Regional Kendall test of significance for annualized data along with a regional estimate of trend (Helsel and Frans 2006). We explored both annual trends and seasonal trends to assess whether precipitation and temperature changes were more distinct during certain seasons. All precipitation data were summed to monthly values and temperature was averaged by month. Temperature was converted to Fahrenheit as the package requires positive input values to get accurate estimates. However, all figures are presented in Celsius.

To specifically address changes in river drying, we estimated trends in flow data from 1980 to 2021 for each metric of drying (e.g., duration of low‐flow) and changes in flow anomalies. We used the non‐parametric Theil‐Sen estimator (i.e., Sen's slope), which is robust to outliers (Sen 1968) using the ‘trend’ R package (Pohlert 2023).

### 2.6 Fish Diversity Trends

For sites with both fish and flow data over the period of record, we explored potential causal relationships on how flow may have contributed to changes in fish diversity. First, we created a species‐by‐time matrix of fish assemblage data for each site. We retained sites with at least 10 years of data for further analysis. These matrices were paired with corresponding flow metrics (including drying metrics and NAA) at the same location (i.e., HexID). Relationships between richness and flow were evaluated using a generalized linear mixed effects model with a Poisson distribution appropriate for count data (i.e., number of species). Year and flow metrics were specified as fixed effects and location as a random effect. To test the effects of flow on changes in species richness over time, we implemented a model selection procedure that included a full model with year and all flow metrics, a null intercept‐only model, and single‐variable models with each year or one of the four flow metrics. Models were evaluated using Akaike Information Criterion (AIC) and model weights. We used the weights to identify a 95% confidence set of models and ratios of Akaike weights. Models within the 95% confidence set, and models within 7 AIC points of each other were considered equally plausible candidate models (Zuur et al. 2009). All variables were centered and scaled to facilitate comparison. Then we evaluated the relationship of time and flow metrics on assemblage composition using a canonical correspondence analysis (CCA) in the ‘vegan’ R package (Oksanen et al. 2024). Canonical correspondence analysis is the most appropriate ordination approach for long gradients that cover a wide spatial or temporal extent (Legendre and Legendre 2012).

### 2.7 Exploring Evolutionary History and Reasons for Conservation Concern in Some Fishes

To evaluate the mechanisms for trends in fish occurrence data over forty years in our dataset, we explored phylogenetic relationships, reasons for being listed on the IUCN, and fish species traits. We categorized fishes as a species of conservation concern if the IUCN Red List indicated they were either extinct, critically endangered, endangered, threatened, or near threatened. As part of our data gathering, we also collated the reason for listing, which included (1) present or threatened destruction, modification, or curtailment of habitat or range; (2) overuse for commercial, recreational, scientific, or educational purposes; (3) disease; (4) other natural or anthropogenic factors affecting persistence (hybridization, exotic or transplanted species, predation, competition); or (5) small range as defined by IUCN.

We tested for phylogenetic relationships that may be related to listing status using the R package ‘FishPhyloMaker’ (Nakamura et al. 2021), which builds a phylogenetic hypothesis (Rabosky et al. 2018) from the species list we provided by pruning species from the ray‐finned fish phylogeny in the ‘fishtreeoflife’ R package (Chang et al. 2019). We then used the ‘phytools’ package (Revell 2012) ‘densityMap’ to fit continuous‐time reversible Markov models to estimate the probability of evolutionary trends describing conservation concern status (0 = not of concern; 1 = species of concern) at each node for 500 simulations. The models assumed equal (0.5/0.5) root node prior probabilities. We plotted the Venn diagram showing reasons for IUCN listing using the ‘ggven’ (Yan 2023) and ‘ggplot2’ (Wickham 2016) packages.

Finally, we ran asymptotic two‐sample Fisher‐Pitman permutation tests in the ‘coin’ package (Hothorn et al. 2006, 2008) to explore if some common traits, including body size, maximum longevity, and trophic level, differed among fish of conservation concern compared to the other xeric fishes in our dataset. This function is analogous to a one‐way linear model but permuted 10,000 times so that the *p*‐value is the proportion of tests with a value at least as extreme as the ‘true’ test. Permutation tests are useful because they are insensitive to data distributions and heteroscedasticity (Helsel 2005).

## 3 Results

### 3.1 Data Extent

Following spatial and habitat‐specific filtering, our final dataset included 40,629 fish occurrence data points from 4495 sampling locations originating from three major data sources (Table 1). Hexagonal spatial binning of these data resulted in a total of 1473 unique sampling locations (hexagons), a majority of which (*n* = 1346) were based in the USA (Figure 1). The number of sample sites per bin ranged from 1 to 34, producing time series of 1 to 41 years in length. Not all hexagons incorporated a stream gauge; 311 hexagons had at least one gauge, with 211 hexagons incorporating multiple (up to 20) gauges (e.g., Figure 1 inset). The high rate of gauges per hexagon was in some cases due to the discontinuation and installation of new gauges in spatially proximate locations, with limited temporal overlap.

### 3.2 Climate Data Trends

**FIGURE 2:**
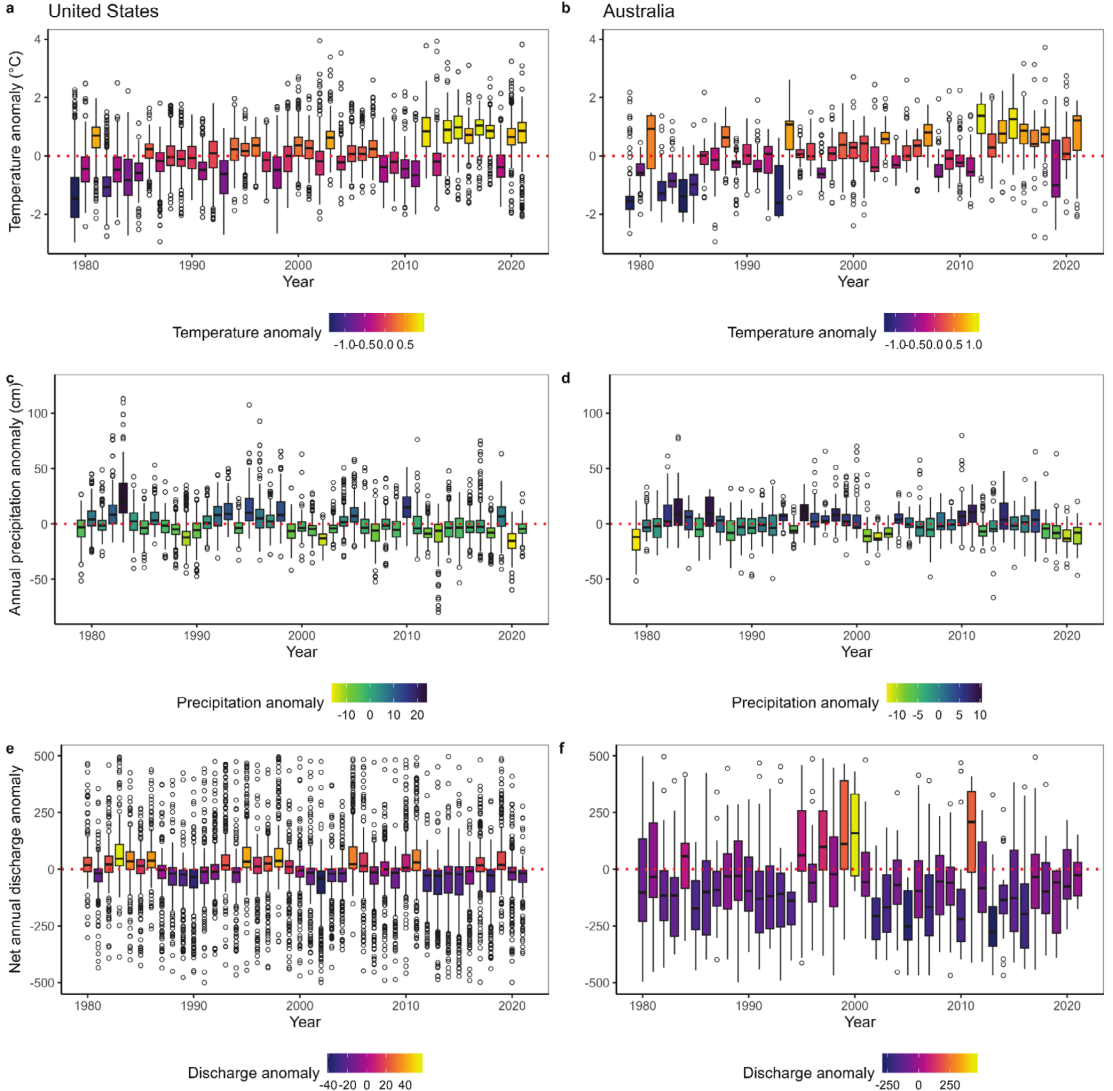
Average annual anomalies in temperature (a, b), precipitation (c, d), and net annual discharge (e, f) in the United States (left panels) and Australia (right panels). Boxplots are filled with the overall median anomaly. Note that the average anomaly scales differ somewhat (e.g., panels e and f). Boxplots depict the minimum, first quartile, median, third quartile, and maximum, with outliers depicted as single points.

Temperatures have warmed at our study sites in both AUS and the USA across seasons and annually (Figures 2a,b and S1; Table S1). Regional Kendall tests indicated strong support for a positive Thiel‐Sen annual temperature slope trend (Sen slope = 0.057° F/year, *p* < 0.00001) in AUS and the USA (Sen slope = 0.052° F/year, *p* < 0.00001). All seasons had positive Thiel‐Sen temperature trends, indicating that warming is not isolated to any portion of the year but is consistent across seasons (Table S1; Figure S1). Temperatures in the USA increased most in the summer season (Figure S1c) with a Thiel‐Sen slope trend of 0.078° F/year, roughly double the slope of increases in other seasons (Table S1; Figure S1c). In contrast, AUS had the largest Thiel‐Sen slope in winter (slope = 0.074° F/year; Table S1; Figure S1h).

Concomitant with temperature, precipitation patterns have changed in both countries (Figures 2c,d and S2; Table S2). Regional Kendall tests on the data indicated strong support for negative annual Thiel‐Sen slope trends (Sen slope = −0.083 mm/year, *p* < 0.0001) in AUS and the USA (Sen slope = −0.137 mm/year, *p* < 0.0001), indicating long‐term decreases in precipitation. Every season had decreased precipitation (Table S2, all *p* < 0.0001) with the exception of summer precipitation in AUS. Average annual total precipitation and variability were similar between xeric regions of the USA (avg = 406 mm, SD = 233) and AUS (avg = 416 mm, SD = 195; Figure S2a,b). In both regions, deficits in precipitation relative to average became more common (Figure S2c,d) – this is especially apparent in AUS where the last 4 years of our time series were well below long‐term averages (Figure S2d). Concurrent with the precipitation decreasing, there were consistent increases in the number of days without any precipitation each year (Figure S2e,f) and the USA had an average of 207 ± 87 (SD) zero precipitation days per year and AUS had 179 ± 87 (SD). On days that did have rain, precipitation intensity increased (Figure S2g,h). Many of the changes in precipitation have happened since 2007 (Figure S2). The average number of days without precipitation increased rapidly in the last 14 years of our study, by 40 days in the USA (avg since 2007 = 247 days) and 29 days in AUS (avg since 2007 = 208 days).

Significant changes to drying metrics in intermittent streams were indicated by the flow data in the USA and AUS (Figure 2e,f, Table 2). There were no significant trends in discharge anomalies over the period of 1980 to 2021. In the USA, there has been a significant increase in the annual number and maximum duration of days with zero flow and no trend in the timing of the onset of low‐flow conditions (Table 2). This indicates that streams which periodically cease to flow in xeric regions of the USA have become more intermittent. In AUS, the annual number and maximum duration of days with zero flow have declined, but the onset of low‐flow conditions has trended toward earlier in the year (Table 2). This indicates intermittent streams in xeric regions of AUS have become wetter, although these trends may be driven by a cycle of several anomalous high flow years near the end of the period of the flow record (Figure 2f).

**TABLE 2:** Sen's slope of trends in stream discharge from 1980 to 2021 in the two focal countries; data include the mean slope, 95% confidence intervals, *p*‐value, and number of gauges (*n*).

Country	Metric	Sen slope	Lower 95% CI	Upper 95% CI	*p*	*n*
USA	NAA	−0.99	−3.33	1.12	0.960	105^a^
	**Zero flow days**	**0.53**	**0.32**	**0.73**	**0.008**	**162** ^b^
	First day minimum flow	0.034	−0.038	0.46	0.227	162^b^
	**Max duration of no flow**	**0.62**	**0.38**	**0.87**	**0.004**	**162** ^b^
AUS^c^	NAA	1.48	−3.39	5.75	0.490	34
	Zero flow days	−0.95	−2.97	1	0.100	34
	**First day minimum flow**	**−0.23**	**−1.42**	**0.19**	**0.048**	**34**
	Max duration of no flow	−1.12	−2.58	0.37	0.100	34

*Note:* Bold metrics had statistically significant *p*‐values.

Abbreviation: NAA, net annual anomaly in discharge.

^a^ HexIDs with fish occurrence and 30 or more years of continuous flow data.

^b^ HexIDs with fish occurrence and gauges that record zero flow.

^c^ Used all gauges, due to low sample size of HexIDs with fish.

### 3.3 Trends in Fish Assemblages

Long‐term, consistent, and repeated fish survey data were rare. In AUS, no location had at least 10 years of data and therefore AUS had no analyses of long‐term trends in fish assemblages. In the USA, 23 locations had at least 10 years of fish survey and corresponding flow data to test relationships between flow metrics and fish richness trends over time.

**FIGURE 3:**
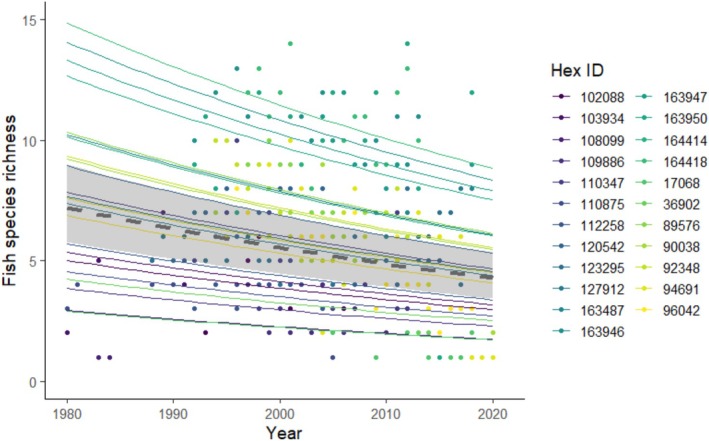
Richness trends through time for all 23 HexIDs with 10 or more years of fish and flow data in the USA. Points represent observed data and lines are model predicted trends over time for the top model, which included year as a predictor (Table 3). The gray dashed line and shaded area represent the overall mean trend and 95% confidence interval. Boxplots depict the minimum, first quartile, median, third quartile, and maximum, with outliers depicted as single points.

Over the period of study, fish richness declined across locations in the USA; however, there was no evidence that these declines were correlated to changing flow conditions, though they were significant through time (Figure 3). There was no relationship between fish richness and normalized flow NAA (*p* = 0.64) nor any of the flow metrics including number of zero flow days (*p* = 0.70), first day of minimum flow (*p* = 0.76), nor max duration of no flow (*p* = 0.57) in a full model containing all the variables. In addition, the difference between models with any single flow metric and a null intercept model was less than 7 AIC, indicating that any individual flow metric was no better at predicting fish richness over a null model (Table 3). In fact, the model containing year as a single factor was 22 times more likely than the full model to explain variation in fish richness, according to the evidence ratio of AIC weights, and the only model retained as a candidate model. Likewise, only 4.5% of the variation in fish assemblage composition could be explained by the combination of year and flow metrics as indicated by the CCA (Total inertia = 11.14; Constrained inertia = 0.50). However, the first axis of the model was significant, with variation primarily driven by year (*χ* ^2^ = 0.33, *p* = 0.001) and total number of low‐flow days (*χ* ^2^ = 0.0731, *p* = 0.022; Figure S3).

**TABLE 3:** The ranked set of models evaluating the effect of flow metrics on fish species richness at a HexID location.

Model	AIC	deltaAIC	Weight
Year	1413.8	0	0.9541
Full model	1420.0	6.2	0.04300
Intercept only	1427.2	13.4	0.0012
Max no flow duration	1429.0	15.2	0.0005
NAA	1429.2	15.4	0.0004
No flow days	1429.2	15.4	0.0004
First day of zero flow	1429.2	15.4	0.0004

*Note:* Akaike's information criteria (AIC), difference in AIC from the top model (deltaAIC), and Akaike weights (weight) for all the models are included.

Abbreviation: NAA, net annual anomaly in discharge.

### 3.4 Fishes of Conservation Concern

**FIGURE 4:**
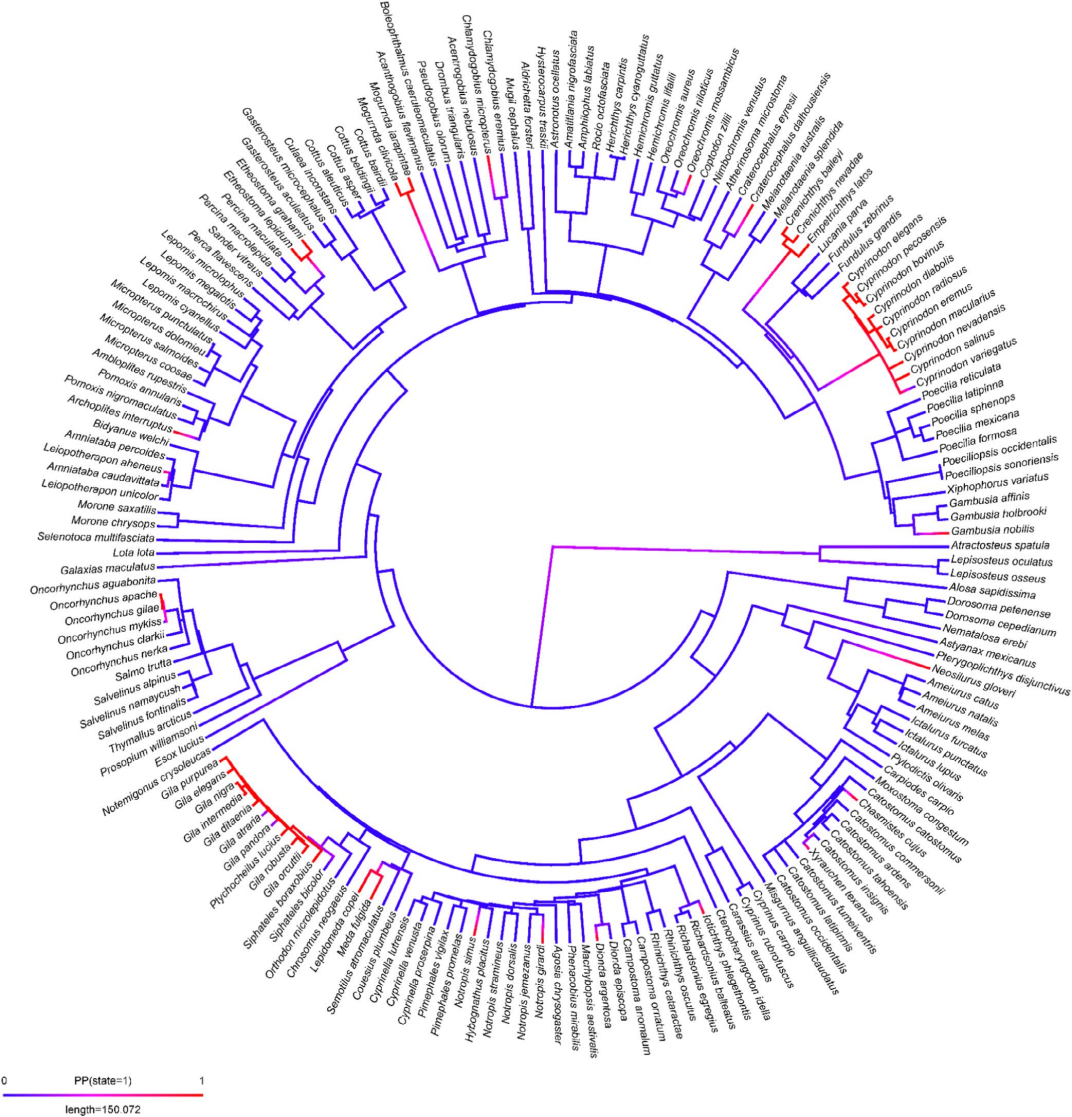
Examination of phylogenetic patterns as a proxy for traits of fishes that occur in xeric ecoregions, which might lead to a species being listed for conservation concern. We simulated the probability of ancestral nodes predicting current conservation status. There were no distinct phylogenetic patterns predicting whether or not a species was of conservation concern. The mapped value (blue = not a species of concern, red = species of concern) is the probability of being in a state 1 (species of concern). Purple indicates a transition between states.

We found a larger proportion of xeric fishes were endangered and extinct compared to general worldwide freshwater stream fishes (Figure S4). Out of 183 fishes within our data, 42 were of conservation concern. Five fish species were near threatened, 15 were vulnerable, 16 species were endangered, and 6 were critically endangered. However, there were no apparent differences between general freshwater stream fishes and those in xeric streams in maximum longevity, size, or trophic level (Figure S5). Furthermore, there were no apparent phylogenetic patterns among xeric fishes of conservation concern (Figure 4). Although certain clades were more likely to be of conservation concern (e.g., *Gila* complex, *Leipidomeda* spp., *Cyprinodon* spp. [red in Figure 4]), these were not linked to phylogenetic relatedness.

**FIGURE 5:**
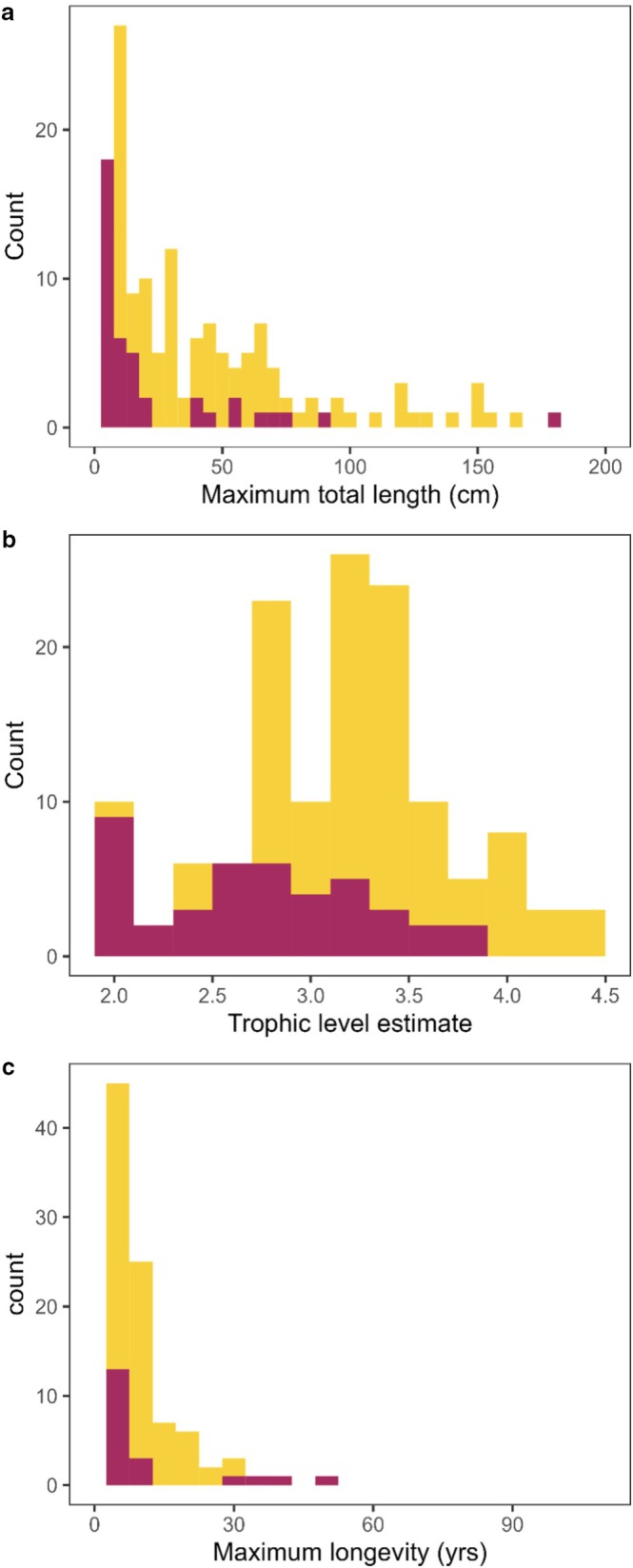
Comparison of (a) maximum total length (in cm), (b) trophic level estimates, and (c) maximum longevity between all of the xeric fishes in our database (yellow) and those that are of conservation concern (red) as listed on the International Union for Conservation of Nature and Natural Resources (IUCN) red list.

**FIGURE 6:**
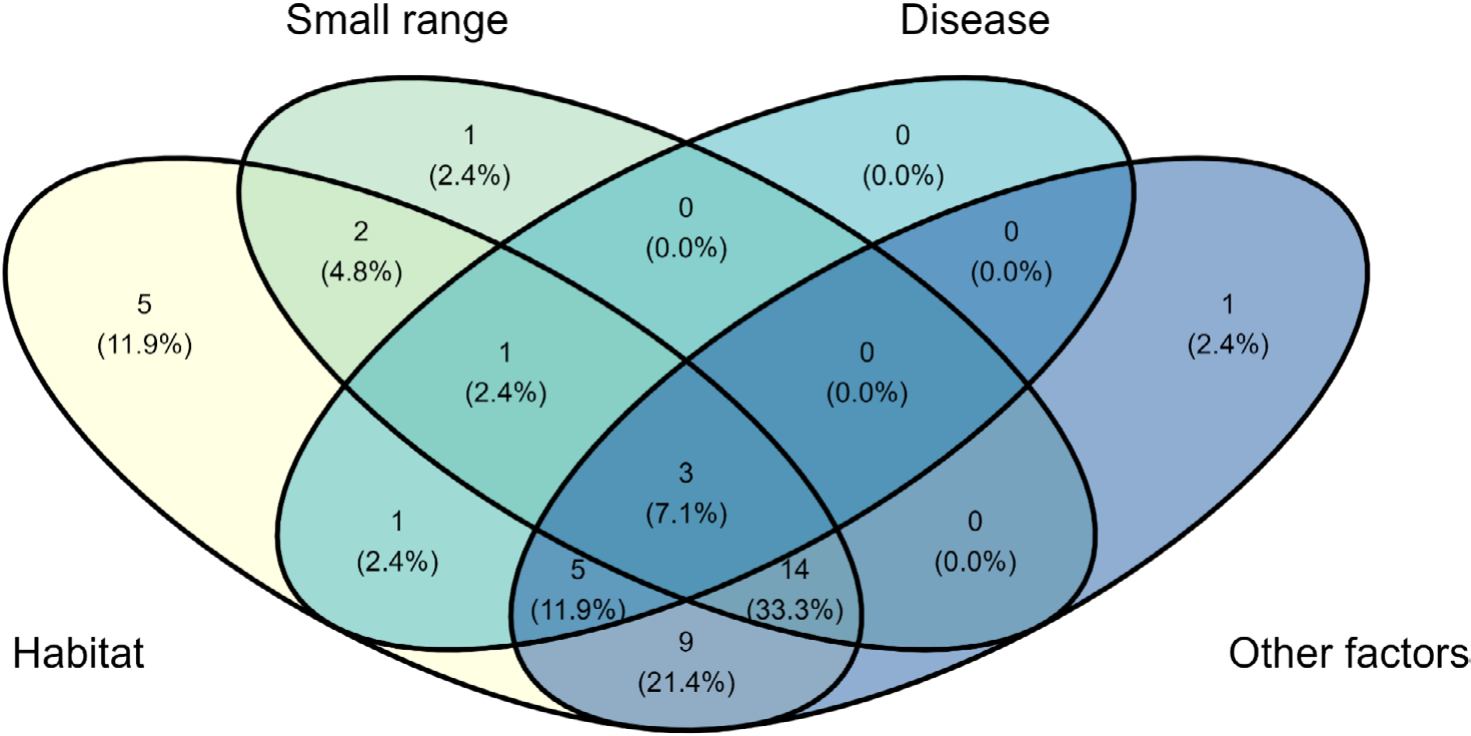
Venn diagram of the top reasons for xeric fishes in our database being listed as either extinct, critically endangered, endangered, threatened, or near threatened. Full definitions: Habitat = “Present or threatened destruction, modification, or curtailment of habitat or range”; Other factors = “Other natural or anthropogenic factors affecting persistence (hybridization, exotic or transplanted species, predation, competition).” No fishes in the database were listed because of overuse for commercial, recreational, scientific, or educational purposes.

Interestingly, two specific traits showed evidence of being related to listing status. Permutation tests indicated xeric fishes of conservation concern tended to have a smaller body size (*Z* = 2.72, *p* = 0.006, Figure 5a) and lower trophic levels (*Z* = 3.83, *p* = 0.0001, Figure 5b) than unlisted xeric fishes. Longevity (*Z* = 0.50, *p*‐value = 0.614, Figure 5c) was not statistically associated with conservation concern listing status. Rather unsurprisingly, myriad stressors are listed as reasons for IUCN listing among xeric fishes (Figure 6). Seven species had only one listing reason, meaning more than 80% of xeric fishes were listed because of multiple threats to persistence. Habitat modification was the primary reason for listing for 38/42 fishes (90%). The next highest ranked reason was “Other natural or anthropogenic factors affecting persistence (hybridization, exotic or transplanted species, predation, competition)” with 32/42 or 76% experiencing this group of stressors. Small range was listed as a reason for listing in 21/42 (50%) of listed xeric fishes, and disease in 10/42 (24%). None of the fishes in our database were listed because of overuse for commercial, recreational, scientific, or educational purposes.

## 4 Discussion

Xeric stream fishes are in danger. A high degree of endemism combined with living at thermal and stream flow extremes means changing climate is likely to have a strong effect on fishes inhabiting xeric streams, especially in combination with other stressors (Comte et al. 2014; Perkin et al. 2021). Our dataset is one of the largest efforts to date to look for global patterns in xeric fishes. Although we found broad‐scale declines in species richness matching our hypothesis, it was not necessarily connected to changing stream flow conditions in the United States, and the lack of publicly available data precluded investigation in Australia. This result was surprising given that flow alteration is implicated as one of the major drivers in fish declines in regional examinations of xeric climates (Perkin et al. 2017) and that increasing aridity and stream intermittency are well documented in the western deserts of the United States (Zipper et al. 2021). However, we did find trends of increasing temperature, decreasing precipitation, and longer duration of no‐flow periods across the xeric ecoregion in both the United States and Australia. Although we found no direct connection between these trends and changes in the fish assemblages we investigated, site‐specific analyses of mechanisms, which are difficult with analyses of similar spatio‐temporal scales to that of this study, may provide a more detailed assessment of potential relationships. Most of the imperiled fishes in our database had multiple stressors leading to listing, most commonly habitat modification, anthropogenic factors or introduced species, and small range, indicating that a combination of stressors is likely a cause for conservation concern among this group of organisms. While we highlight important patterns in our findings, we acknowledge that the use of large, publicly available datasets precludes our ability to make high‐resolution conclusions about individual assemblage responses to climate change. We suggest our analysis of large‐scale trends is a useful springboard for examination of trends at local scales where greater resolution is possible.

### 4.1 Climate Is Changing

We found strong and consistent patterns of increasing temperature and decreasing precipitation in both the United States and Australia, which likely affect both physiological and food web processes. It is getting hotter across all seasons, and this trend is especially apparent since 2010. For xeric fishes already living at the extremes of thermal tolerances, these increases could push streams to lethal temperature levels and give more temperature‐tolerant non‐native fishes a competitive advantage (Archer and Predick 2008). These warmer temperatures are combining with changing precipitation regimes in a manner that will likely make xeric streams more intermittent and physiologically challenging environments. Precipitation is not only decreasing across almost all seasons and regions, but the average number of days per year with zero precipitation is increasing on both continents along with precipitation intensity. Less frequent but more intense precipitation events are concerning given that droughts followed by intense rain can result in fish kills (Whitworth et al. 2012). Climate change is also apparent in the discharge data. Our analyses show increases in the number of zero flow days and duration of no flow in the United States and earlier onset of minimum flow in Australia. Considering that gauging stations are disproportionately located in areas with consistent flow (Krabbenhoft et al. 2022), this is likely an underestimate of actual stream flow changes. The shifting climate conditions we document are highly likely to expand the spatial extent of intermittency. Based on stream network geography, many species have limited avenues to escape increasing temperatures and intermittency regimes (Hermoso et al. 2013; Kovach et al. 2019). Approximately 90% of the fishes of conservation concern in this study were listed in the IUCN due to “present or threatened destruction, modification, or curtailment of habitat or range.” These species exist in environments where humans develop riparian land and construct dams, further affecting flow regimes (Li and Quiring 2021). The combination of habitat loss with altered temperature and precipitation regimes could move many of these taxa beyond a stressor threshold that would allow populations to persist (Mantyka‐pringle et al. 2011). We thus suggest that fish assemblages in xeric streams may warrant additional monitoring and management to aid in long‐term conservation strategies.

### 4.2 Species Richness Is Changing

Although our approach is focused on broad‐scale patterns and data are limited for high‐resolution conclusions, we did find long‐term declines in fish species richness at the 23 locations with 10 or more years of fish and flow data in the United States. Although this trend is concerning, we cannot assume native species loss is pervasive across xeric ecoregions due to the small sample size of the dataset, the paucity of complementary fish and flow data from Australia, and the lack of publicly accessible data from other nations with xeric ecoregions. Furthermore, these findings highlight the importance of monitoring and long‐term datasets for discovering trends, identifying causal linkages, and generating alternative explanations to pursue further research and monitoring to ultimately inform potential conservation and management actions. The most effective strategies for tackling conservation strategies in the future will depend explicitly on the individual causes for decline (Paukert et al. 2021), thus necessitating research into species‐ and system‐specific trends. For the United States dataset, variability in discharge and intermittency had insufficient explanatory power to describe the overall negative trend in fish species richness, nor were they correlated with changes in assemblage composition. However, climate‐associated stream effects are not limited to flows. Warming temperatures and changes in precipitation and evaporation may lead to direct effects on fish physiology, which subsequently influence the survival, reproduction, and distribution of species (Paukert et al. 2021). For example, lower precipitation and higher evapotranspiration rates have increased the salinity of some rivers in xeric regions and created prominent changes in species composition (Miyazono et al. 2015). Furthermore, the effects of climate change can exacerbate other environmental and anthropogenic stressors to rivers including increasing water demands, urban development, channelization, barriers, and harmful invasive species (Dudgeon 2019). There may also be some effect of dams or other impoundments altering hydrologic signals of climate change, contributing to alternative changes in stream flows and habitats (e.g., dampened seasonality and magnitude of flows, cooler water temperatures, higher evaporation rates, and altered water chemistry; Shakarami et al. 2022). Individually, or cumulatively, these factors may have influenced the observed biodiversity declines, but identifying mechanistic relationships will require further study and long‐term datasets, particularly in locations like Australia with large areas of xeric lands, but relatively few publicly available fish occurrence datasets. The observed changes in richness imply that in order to preserve the long‐term persistence of xeric stream fishes, attention to multiple and interacting stressors warrants consideration within conservation frameworks.

### 4.3 Species, Traits, and Endemism

Xeric fishes have adapted reproduction strategies that maximize resource availability at the time of spawning and may even enhance recruitment in response to projected resource availability (Turner et al. 2010). For example, studies in both the United States and Australia have shown shifting spawning phenologies in response to variation in hydrologic conditions (Krabbenhoft et al. 2014; Tyler et al. 2021). Despite these adaptations, changes in temperature and precipitation phenology can disrupt reproductive cues (Acre et al. 2023). Greater ability to disperse and shift distributional range allows fishes to persist under these conditions (Jaeger et al. 2014). However, fishes' abilities to shift their distribution are in part a function of their diet breadth, with a lower tendency for more specialized feeders to track climate shifts (Whitney et al. 2017). Global climate change is also likely to affect resource subsidies between aquatic and terrestrial ecosystems (Larsen et al. 2016). Model efforts indicate warmer, drier conditions result in 30 to 40% reductions in macroinvertebrate taxa (Pyne and Poff 2016) and drying can lead to remarkable changes in riparian arthropod communities, which can be important resources for stream fishes (Allen et al. 2014, 2024). Although xeric fishes have evolved mechanisms for living at the extremes, some of these same adaptations that allowed them specificity to xeric streams may prove disadvantageous with the compounding effects of anthropogenic stressors and the disruption of climate regimes.

Twenty‐three percent of the fishes in our data are listed as IUCN species of concern, with more than 50% of our listed species of concern because of small geographic range. Many of these fishes are endemic, including species such as the Apache trout ( *Oncorhynchus apache* ), Gila trout ( *O. gilae* ), Gila chub, and Rio Grande darter ( *Etheostoma grahami* ). Although we did not find any phylogenetic patterns in whether or not a fish species was vulnerable, xeric fishes of conservation concern had small body sizes, matching previous research (van der Lee et al. 2015), and occupied low trophic levels. We additionally had many cosmopolitan species (e.g., *Gambusia* sp.) as well as stocked and/or invasive piscivores in our database (e.g., *O. mykiss* , *Salmo trutta* , *Micropterus dolomieu* ). Increasing drought can favor nonnative fishes (Rogosch et al. 2019) and nonnative piscivores are particularly adept at range expansion with climate change (Whitney et al. 2017). Xeric fishes are more sensitive to interannual variation in climate than non‐native fishes (Gido et al. 2019), and small body size tends to limit the ability to disperse (Olden et al. 2006). For example, naturally small geographic ranges and non‐native introductions have been pervasively linked to conservation status in the Chihuahuan desert (Perkin et al. 2021). Nonnative piscivores are associated with native fish species declines in some xeric streams (Whitney et al. 2014), and small‐bodied fish would be more vulnerable to predation. Changes in xeric streams can favor nonnative fishes that act as competitors and predators, putting more pressure on xeric species. This matches the second most common reason for conservation concern listing, “other natural or anthropogenic factors affecting persistence (hybridization, exotic or transplanted species, predation, competition).” Together, increased climate stochasticity and nonnative fishes may interact to drive larger changes in xeric fish assemblages than either factor alone (Rogosch and Olden 2019; Ruhí et al. 2016). Further, xeric streams may be moving toward biotic homogenization, whereby introduced generalist species combined with the extirpation of endemic species increases the similarity among regional fish assemblages (Olden and Rooney 2006). The relatively high proportion of endemic fishes in our dataset may thus make assemblages more vulnerable to anthropogenic stressors, including invasion and biotic homogenization. Additional data from xeric streams that exist in areas with lower human population densities than more mesic areas (Taylor 2002) would provide a valuable framework for comparisons to test these hypotheses.

### 4.4 Conclusions

Global analyses are only made possible by the long‐term, consistent efforts of those dedicated to sampling in some of the most extreme freshwater environments. Investment in repeated, long‐term research and the effort to make the data accessible to the public, researchers, and policymakers is paramount to understanding how the phylogenetic, phenotypic, and physiologic uniqueness of fishes will be affected by anthropogenic and climate stressors. These investments in data and research would provide fundamental information on status and trends to aid the conservation of xeric fishes. Despite their evolutionary history and suites of traits that contribute to the ability to persist in xeric environments, the rate of change incurred in a modern climate poses a conundrum for even the fish best adapted to xeric streams. Our analysis highlights the role of habitat loss as a critical factor in the persistence of these fishes, and when combined with a myriad of other stressors, the environmental context of modern xeric streams may move beyond the limits of native species to respond. If fishes that are specifically adapted to these environments cannot keep up with the physiological demands of modern climate change and other compound stressors, similar concerns for species less adapted to shifts in hydrologic extremes and drier conditions may follow. Although conclusions in Australia are limited due to a paucity of long‐term fish occurrence data, we illustrate similar patterns in climate and listing status in the United States and Australia, highlighting the global nature of these patterns. As harbingers of changes to come, our monitoring and understanding of xeric fishes is critical to informing the protection of regional and global diversity in freshwater systems.

## Author Contributions

**Corey A. Krabbenhoft:** conceptualization (equal), data curation (equal), formal analysis (equal), investigation (equal), methodology (equal), visualization (equal), writing – original draft (equal), writing – review and editing (equal). **Jane S. Rogosch:** conceptualization (equal), data curation (equal), formal analysis (equal), investigation (equal), methodology (equal), visualization (equal), writing – original draft (equal), writing – review and editing (equal). **Freya E. Rowland:** conceptualization (equal), data curation (equal), formal analysis (equal), investigation (equal), methodology (equal), visualization (equal), writing – original draft (equal), writing – review and editing (equal).

## Conflicts of Interest

The authors declare no conflicts of interest.

## Supporting information


**Appendix S1:** Supporting Information S1.

**Appendix S2:** Supporting Information S2.

## Data Availability

Data are all publicly available. The DOI for the datasets or a URL for the data storage locations are listed in Supporting Information S2. Any additional questions can be directed to the corresponding author at ckrabben@buffalo.edu.
