# Supplementary material for: Long‐Term Regime Shifts in Xeric Ecoregion Freshwater Fish Assemblages due to Anthropogenic and Climate Stressors
Source: Ecol Evol. 2025 Sep 1;15(9):e72067. doi: 10.1002/ece3.72067 (PMC12401659; doi:10.1002/ece3.72067)
Supplement: Supplementary file 1 — Appendix S1: Supporting Information S1. [file ECE3-15-e72067-s002.docx]

**Long-term regime shifts in xeric ecoregion freshwater fish assemblages due to anthropogenic and climate stressors**

Corey A. Krabbenhoft^1*^, Jane S. Rogosch^2^, Freya E. Rowland^3^

^1^Department of Biological Sciences, Hochstetter Hall 609, University at Buffalo North Campus, Buffalo, NY 14260; ORCID: 0000-0002-2630-8287

^2^U.S. Geological Survey, Texas Cooperative Fish & Wildlife Research Unit and Department of Natural Resources Management, Texas Tech University, 1312 Boston Ave, Lubbock, TX 79409

^3^U.S. Geological Survey, Columbia Environmental Research Center, 4200 New Haven Rd, Columbia MO 65201; ORCID: 0000-0002-1041-5301

^*^Corresponding author: ckrabben@buffalo.edu

# Supporting Information S1

**Table S1.** Regional Seasonal Kendall test results fit using the ‘wql’ package (Jassby and Cloern 2022) to examine how regional average monthly temperature has changed across seasons from 1980-2021. Seasons were defined as 1 December - 28/29 February = summer (AUS) or winter (USA); 1 March - 31 May = fall (AUS) or spring (USA); 1 June - 31 August = winter (AUS) or summer (USA); 1 September – 30 November = spring (AUS) or fall (USA). Temperature was scaled from Celsius to Fahrenheit because the model cannot handle negative values.

| Season | Climate variable | Site | Sen slope | p-value |
| --- | --- | --- | --- | --- |
| Annual | temperature | Australia | 0.057 | <0.0001 |
|  | temperature | USA | 0.052 | <0.0001 |
| Spring | temperature | Australia | 0.056 | <0.0001 |
|  | temperature | USA | 0.039 | <0.0001 |
| Summer | temperature | Australia | 0.045 | <0.0001 |
|  | temperature | USA | 0.078 | <0.0001 |
| Fall | temperature | Australia | 0.045 | <0.0001 |
|  | temperature | USA | 0.044 | <0.0001 |
| Winter | temperature | Australia | 0.074 | <0.0001 |
|  | temperature | USA | 0.031 | <0.0001 |

**Table S2.** Regional Seasonal Kendall test results fit in the ‘wql’ package (Jassby & Cloern, 2024) using the to examine how regional precipitation (in mm per month) has changed across seasons from 1980-2021. Seasons were defined as 1 December - 28/29 February = summer (AUS) or winter (USA); 1 March - 31 May = fall (AUS) or spring (US); 1 June - 31 August = winter (AUS) or summer (USA); 1 September - 30 November = spring (AUS) or fall (USA).

| Season | Climate variable | Site | Sen slope | p-value |
| --- | --- | --- | --- | --- |
| Annual | precipitation | Australia | -0.083 | <0.0001 |
|  | precipitation | USA | -0.137 | <0.0001 |
| Spring | precipitation | Australia | -0.045 | <0.0001 |
|  | precipitation | USA | -0.186 | <0.0001 |
| Summer | precipitation | Australia | 0.007 | 0.699 |
|  | precipitation | USA | -0.055 | <0.0001 |
| Fall | precipitation | Australia | -0.112 | <0.0001 |
|  | precipitation | USA | -0.164 | <0.0001 |
| Winter | precipitation | Australia | -0.118 | <0.0001 |
|  | precipitation | USA | -0.066 | <0.0001 |


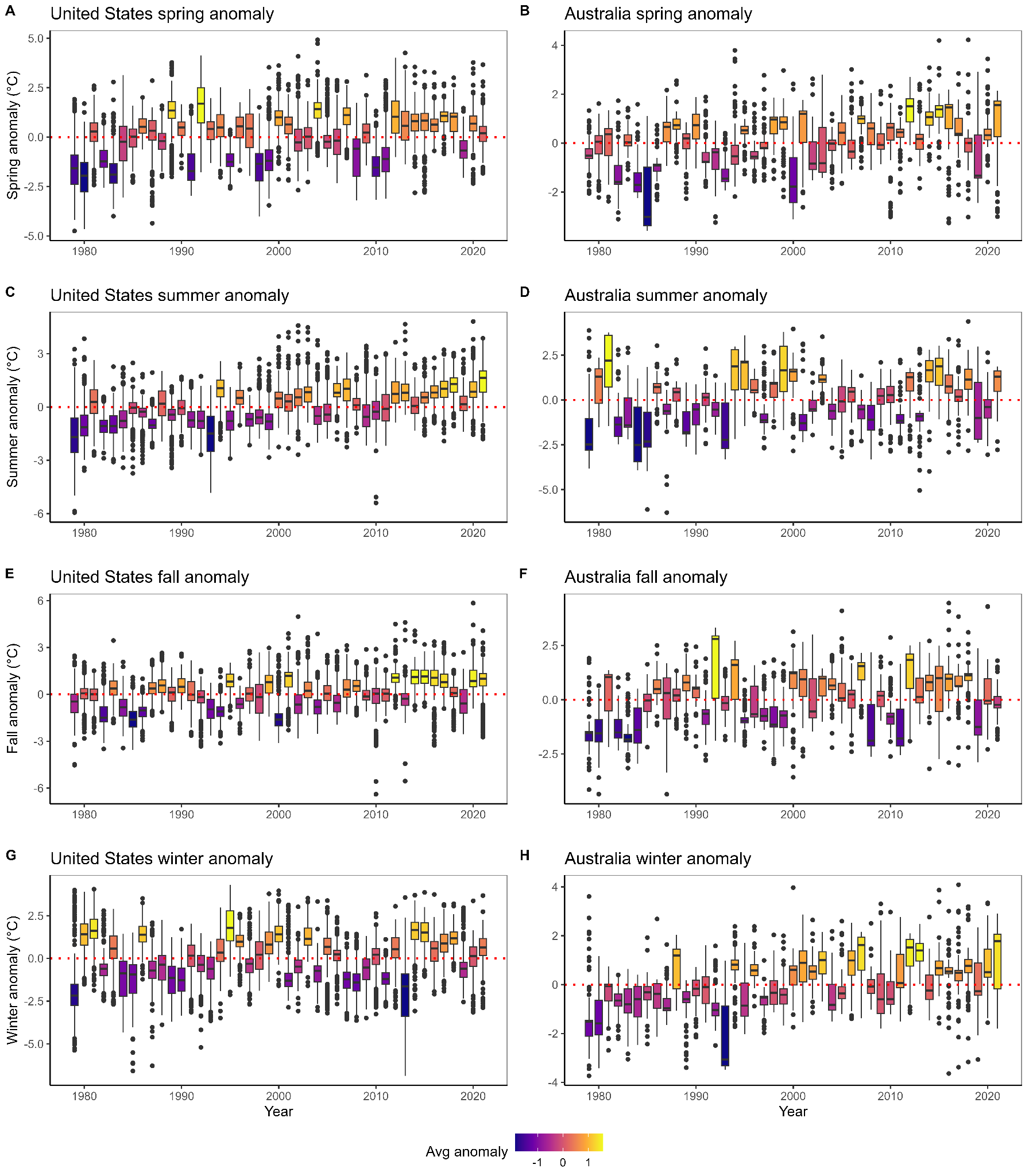


**Figure S1.** Temperature anomalies in the United States (A,C,E,G) and Australia (B, D, F, H). Anomaly was calculated for each station (n = 1327 United States; n = 118 Australia) as the long-term average for that season minus the average within a year. Box plots depict the minimum, first quartile, median, third quartile, and maximum, with outliers depicted as single points.


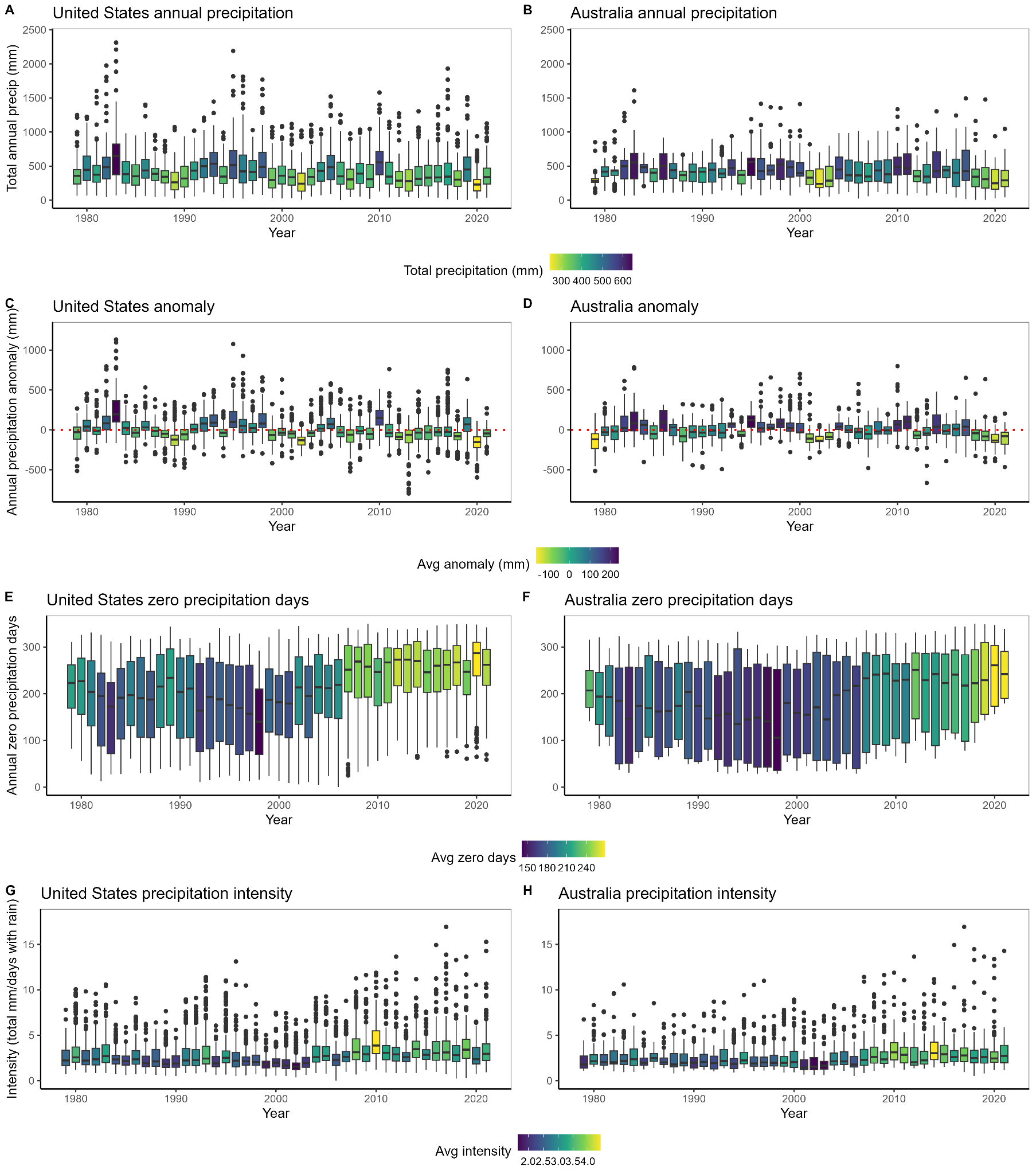


**Figure S2.** Precipitation trends including (**A, B**) annual precipitation totals (mm); (**C, D**) precipitation anomaly defined as precip_i_ - precip_avg_**;** (**E, F**) annual number of days with zero precipitation; and (**G, H**) precipitation intensity defined as annual precipitation total / number of days with precipitation. Precipitation data were from n = 1335 stations in the United States and n = 40 stations in Australia. Data are presented as boxplots color-coded by overall average among all stations within a year. Box plots depict the minimum, first quartile, median, third quartile, and maximum, with outliers depicted as single points.


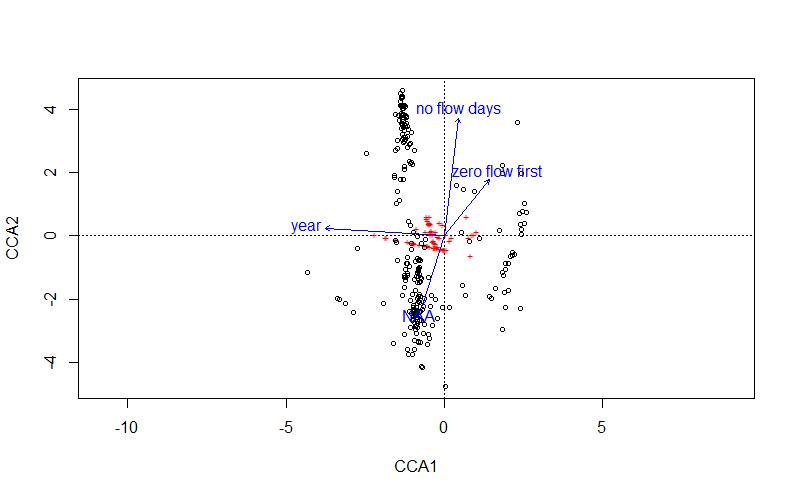


**Figure S3.** Ordination bi-plot of presence/absence of fish species at 23 locations in the United States with at least 10 years of data between 1980 and 2021, related to time and flow metrics. The species are represented by plus symbols (red) and sites by open points (black). Axis I: 3.2% of the variation (p = 0.001) after 999 permutations; Axis II: 0.8% of the variation (p = 0.08). CCA = canonical correspondence analysis.


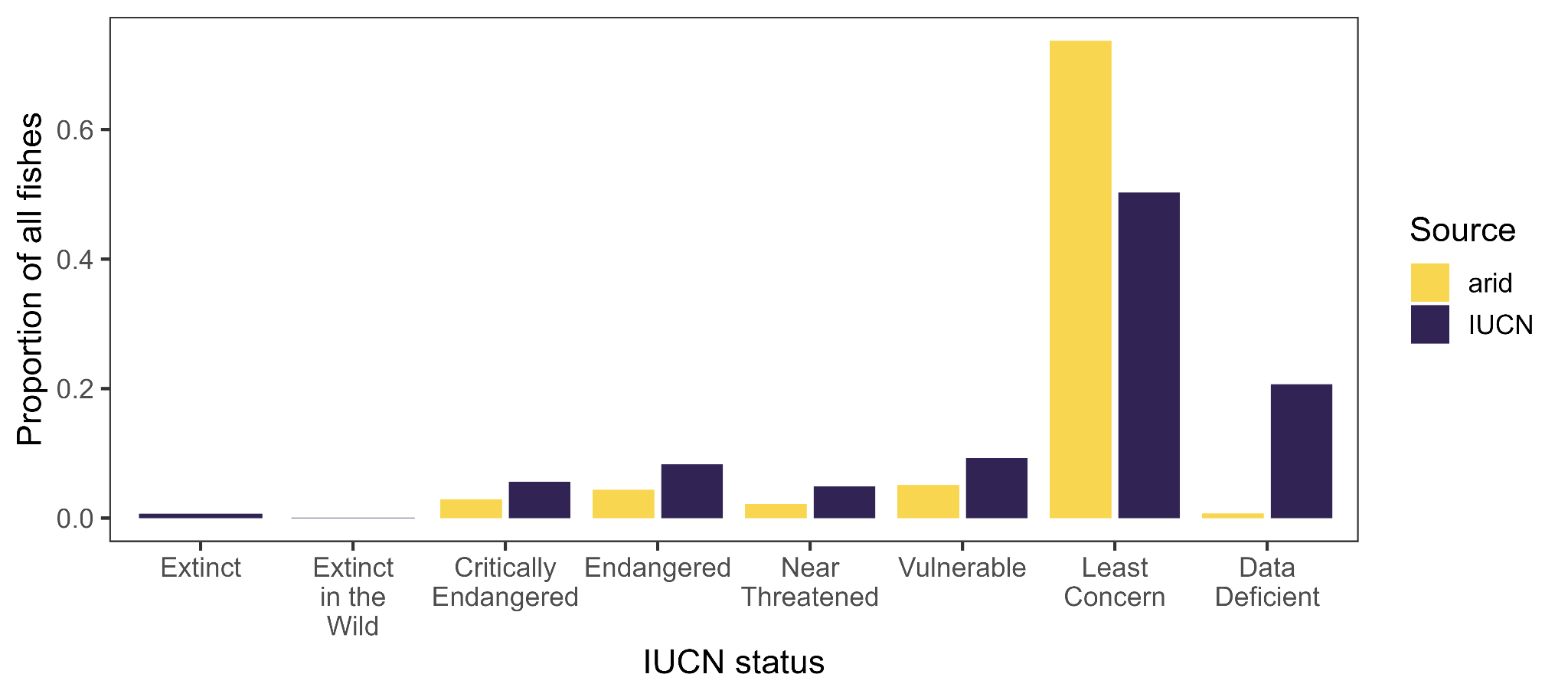


**Figure S4.** Comparison of International Union for Conservation of Nature and Natural Resources (IUCN) status of global stream freshwater fishes (purple; n = 11,211) and xeric stream fishes (yellow; n = 137). There were no differences between xeric fishes and the larger IUCN stream fish database, although 11% (n = 15) of the xeric fishes in our database have not been evaluated. We downloaded global freshwater fish data from the IUCN website and ggplot2 (Wickham 2016) to summarize.


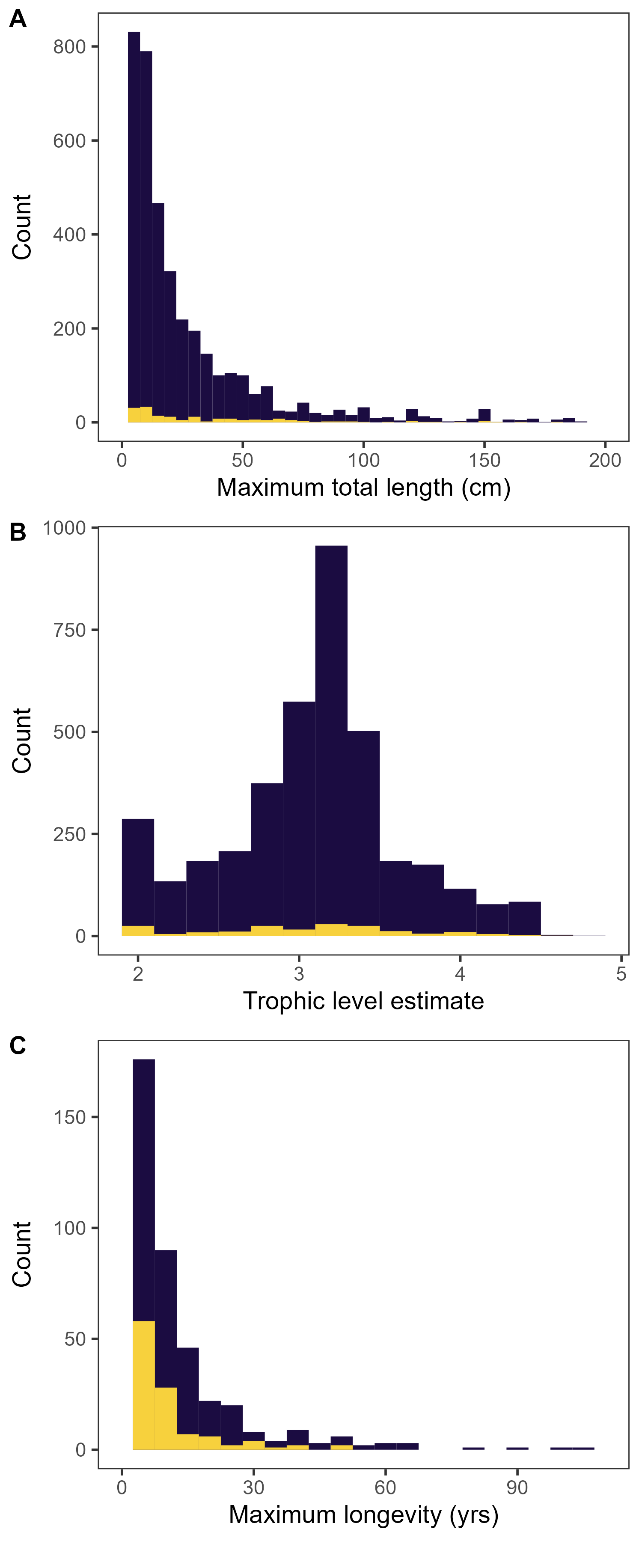


**Figure S5**. Comparison of (**a**) maximum total length (in cm), (**b**) trophic level estimate, and (**d**) maximum longevity (yrs) between freshwater stream fishes in fishbase.org (blue bars) for xeric fishes (yellow). We used the ‘rfishbase’ package (Boettiger et al., 2012) to get stream fish data and ggplot2 (Wickham, 2016) to summarize.

References cites in the Supplement

Boettiger, C., Lang, D. T., & Wainwright, P. C. (2012). rfishbase: exploring, manipulating and visualizing FishBase data from R. Journal of Fish Biology, 81, 2030–2039. <https://doi.org/10.1111/j.1095-8649.2012.03464.x>

Jassby, A. D., & Cloern, J. E. (2024). *wql: Exploring water quality monitoring data* (1.0.0). https://cran.r-project.org/package=wq

Wickham, H. (2016). *ggplot2: Elegant graphics for data analysis*. Springer-Verlag New York. https://ggplot2.tidyverse.org
